# Case Report: A fatal case of CNS actinomycosis mimicking Tolosa–Hunt syndrome: Is it time to reevaluate diagnostic criteria?

**DOI:** 10.3389/fmed.2025.1649779

**Published:** 2025-09-11

**Authors:** Natalie Bristol, Courtney Venegas, Isha Snehal, Mathias Maisenbacher, Ryan Higgins, Thomas Auen, Catherine Cichon, Richard Hankins, Christie Barnes, Sahara Cathcart, Ana Yuil-Valdes, Jie Chen, Cindy Schmidt, Subin Mathew

**Affiliations:** ^1^Department of Internal Medicine, University of Nebraska Medical Center, Omaha, NE, United States; ^2^Department of Neurology, University of Nebraska Medical Center, Omaha, NE, United States; ^3^Neurology, Mayo Clinic Minnesota, Rochester, MN, United States; ^4^Department of Otolaryngology, University of Nebraska Medical Center, Omaha, NE, United States; ^5^Department of Pathology, Microbiology and Immunology, University of Nebraska Medical Center, Omaha, NE, United States

**Keywords:** headaches, painful ophthalmoplegia, non-specific granulomatous inflammation of the cavernous sinus, magnetic resonance imaging (MRI), soft tissue mass involving the cavernous sinus, central nervous system (CNS) actinomyces infection, serologic next-generation sequencing (NGS)

## Abstract

**Background:**

Tolosa-Hunt Syndrome (THS) is a rare diagnosis characterized by headaches, painful ophthalmoplegia, and granulomatous inflammation of the cavernous sinus. It is a diagnosis of exclusion but can mimic many other conditions, including central nervous system (CNS) actinomycosis. This systematic review evaluated the frequency of CNS actinomycosis initially diagnosed as THS and evaluated lapses in the current diagnostic criteria which were related to poor outcomes.

**Case report:**

We present a case of a 43-year-old man who was diagnosed with THS 10 weeks prior to his final hospital presentation. Previous infectious work up was negative. He returned with altered mental status and was found to have leptomeningeal enhancement, vasculitis, and acute infarcts. He decompensated while on broad-spectrum antimicrobials. The patient was ultimately found to have CNS actinomycosis when next generation sequencing identified *Actinomyces israelii*. This diagnosis was confirmed by biopsy. Unfortunately, due to the extent of the infarcts, the patient passed away.

**Results:**

A total of 344 records were analyzed for title/abstract review and ultimately revealed Eight cases of CNS actinomycosis initially diagnosed as THS. 62.5% of cases had dental history prior to symptom onset. Cerebral spinal fluid cultures were obtained in 75% percent of cases, and all were negative for Actinomyces. 88% of cases were diagnosed by histopathology evaluation and 25% of cases were fatal.

**Conclusion:**

With the low reported prevalence of CNS actinomycosis, the inadequacy of current testing for Actinomyces, and the significant overlap of symptoms with THS, it is essential that clinicians are aware of CNS actinomycosis as a potential infectious etiology if early and potentially curative treatment is to be provided. The current definition of THS may encourage harmful practices and should be revised.

## Highlights

- THS is a diagnosis of exclusion; infectious mimics such as *Actinomyces* must be ruled out before initiating steroids.
- NGS can be a useful adjunct in the diagnosis but must be confirmed by tissue sampling.
- Early biopsy and broad empiric antimicrobials in atypical THS or steroid-unresponsive cases may reduce mortality.

## Introduction

Tolosa–Hunt Syndrome (THS) is a rare diagnosis characterized by headaches, painful ophthalmoplegia, and non-specific granulomatous inflammation of the cavernous sinus. Magnetic resonance imaging (MRI) of the brain typically demonstrates a soft tissue mass involving the cavernous sinus (IHS International Classification of Headache Disorders 3) (1). This is considered a diagnosis of exclusion, with a broad differential diagnosis that includes malignant, infectious, and rheumatologic conditions. Central nervous system (CNS) actinomycosis infection is an uncommon and potentially fatal condition that can mimic THS but is difficult to diagnose in the absence of tissue cultures or other microbiologic tests, such as serologic next-generation sequencing (NGS) (2). When working up THS, it is imperative that clinicians rule out CNS actinomycosis prior to proceeding with steroids to prevent the aggravation of a potentially life-threatening infection. In cases of possible THS where the patient or provider wishes to avoid a cavernous sinus biopsy with bacterial cultures, we propose using serologic NGS to help rule out infections before diagnosing THS.

## Background

THS was first described in the year 1954 by a Spanish neurosurgeon Dr. Eduardo Tolosa (3). Similar cases were subsequently described by Hunt et al. (4). According to the ICHD-3 (1), the diagnostic criteria for THS are as follows:

Diagnostic criteria:

A Unilateral orbital or periorbital headache fulfilling criterion C.
B Both of the following:

1 Granulomatous inflammation of the cavernous sinus, superior orbital fissure, or orbit, demonstrated by MRI or biopsy.
2 Paresis of one or more of the ipsilateral third, fourth, and/or sixth cranial nerves.

C Evidence of causation demonstrated by both of the following:

1 Headache is ipsilateral to the granulomatous inflammation.
2 Headache has preceded the paresis of the third, fourth, and/or sixth cranial nerves by ≤2 weeks, or developed with it.

D Not better accounted for by another ICHD-3 diagnosis.

Glucocorticoids have been the standard treatment for THS since the 1960s, despite only a few case studies and series being performed to delineate such guidelines (5). Unfortunately, this has led to variability in the dosage, schedule, and administration of steroid therapy, but often recommended guidelines include a three-day course of prednisone at an 80–100 mg dosage, followed by an approximately eight-week taper thereafter. Glucocorticoids seem to hasten the resolution of orbital pain; however, there is no definitive evidence that cranial neuropathies recover more quickly with treatment than without. Other treatment options, such as immunosuppressants, have not been explored for this entity either, likely related to the fact that pain resolves very quickly with glucocorticoids (6).

The pathogenesis of THS is not fully understood. Although it is known that THS is characterized by granulomatous inflammation that is highly responsive to steroids, no specific etiology has been implicated. Patients often experience relapses, needing multiple courses of immunosuppression. Inflammation and pressure result in secondary dysfunction of the structures within the cavernous sinus (aforementioned cranial nerve palsies). Intracranial extension of the disease process has been reported, although there is no evidence of systemic inflammation, which also makes this process unique (7). Furthermore, up to 75% of patients presenting with painful ophthalmoplegia do not have THS (7). Ultimately, THS is a diagnosis of exclusion.

Actinomycosis is a relatively uncommon infection caused by Gram-positive, anaerobic, non-acid-fast organisms in the *Actinomycetaceae* family. These filamentous bacteria can be found on the mucosal surfaces of the oropharynx, gingiva, gastrointestinal, and genitourinary tracts (8). Although usually commensal, they can become pathogenic upon disruption of the mucosa. These opportunistic organisms make it rather difficult to determine if they are the causal pathogens in some cases. This family of bacteria usually presents as clinically indolent and eventually accumulates into masses, which can form draining fistulas. *Actinomyces israelii* most commonly causes cervicofacial infections (24) but has also been known to cause abdominal, pulmonary, and, very rarely, CNS diseases (9). In 1987, a systematic review published by Smego et al. revealed 70 cases of CNS actinomycosis. A second review was published in 2023 by Meena et al., which evaluated 118 cases from 1988 to 2023. However, the true incidence of CNS actinomycosis is difficult to assess due to its infrequent occurrence and difficult identification. Based on the case series presented by Meena et al. and Ravindra et al., CNS actinomycosis accounts for less than 5% of all actinomycosis cases and approximately 0.6% of all brain abscesses, with a mean presentation of approximately 31–44 years and a male predominance (57–76%; 2023 & 2018). Risk factors include dental procedures, dental infections, facial trauma, otitis media, and mastoiditis (2, 10). The granulomatous inflammatory response of *Actinomyces* can mimic mycobacteria, *Nocardia*, and fungal infections, as well as malignancies and inflammatory conditions such as THS. It is most narrowly treated with penicillin G; however, it is effectively treated with ceftriaxone-based regimens.

## Case report

A 43-year-old man with a pertinent past medical history of opioid use disorder and very recently diagnosed hypertension, otherwise healthy and independent, presented to the emergency department (ED) with a three-week history of headaches, progressive left-sided ophthalmoplegia, and diplopia. He reported gingival lesions several weeks before but did not seek treatment. The initial neurological examination disclosed left oculomotor and abducens palsies. The MRI performed to evaluate for underlying mass lesions showed asymmetrical soft tissue fullness at the left aspect of the sella, with left cavernous sinus involvement. There was also evidence of a left internal carotid artery (ICA) flow void in that region. The MRV performed to evaluate for sinus thrombosis in the setting of elevated intracranial pressure leading to cranial nerve palsies showed no evidence of cavernous sinus thrombosis. Ophthalmology was consulted to rule out primary ocular pathology. Cerebral spinal fluid (CSF) studies—performed to evaluate for underlying infection, autoimmune or inflammatory conditions, or malignancy—showed unremarkable cell counts, a negative meningitis polymerase chain reaction (PCR) panel, and negative bacterial, fungal, and acid-fast bacterial (AFB) cultures. Fungal serologies for *Histoplasma*, *Coccidioides*, and *Blastomyces*, as well as HIV antigen/antibody panel, A1c, *Borrelia burgdorferi* antibody testing, and syphilis antibody testing, were also negative. Cell cytology came back negative for malignancy. Neurosurgery was consulted for biopsy evaluation; however, the patient ultimately declined a biopsy due to fear of surgical complications in the setting of his small lesion near eloquent tissue in the cavernous sinus. The patient met the criteria for THS as outlined in the ICHD-3. Following completion of the recommended eight-week 60 mg prednisone taper, the patient reported significant improvement in ocular symptoms, although headaches persisted.

**Figure 1:**
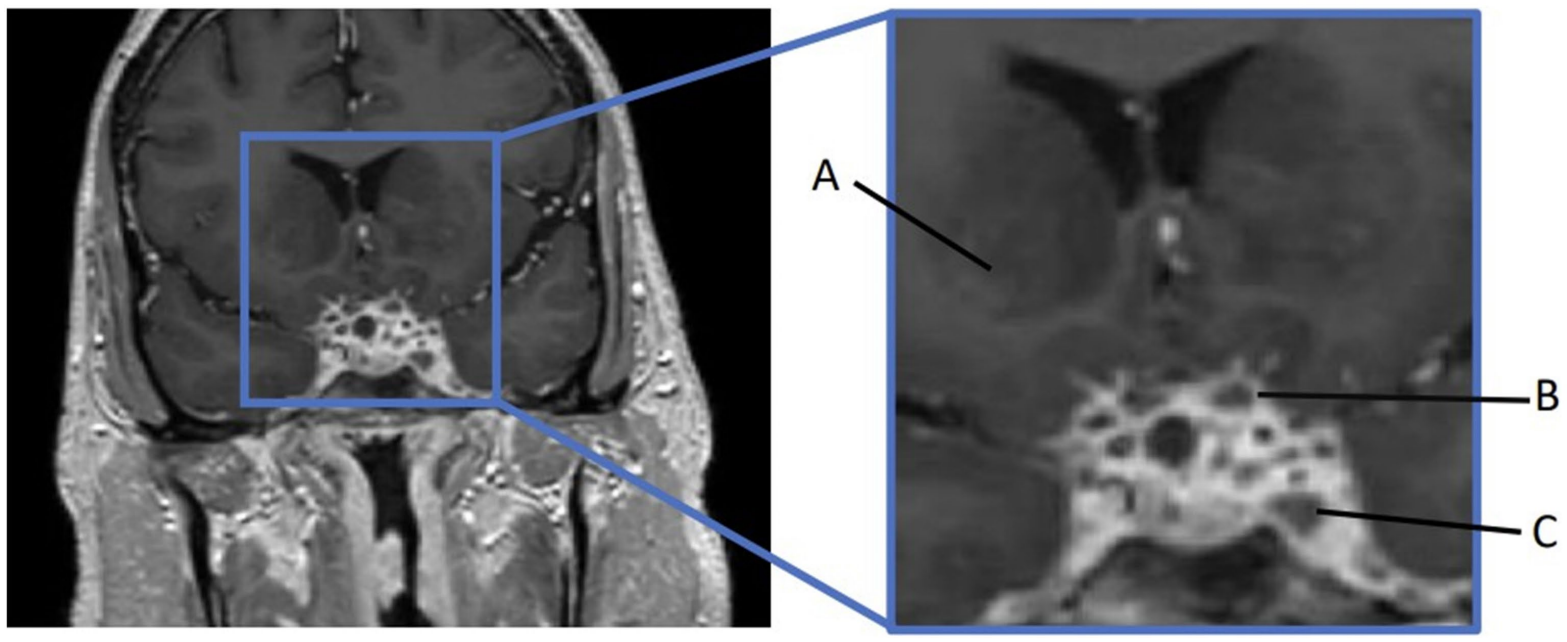
MRI skull base T1 + C at 6-week follow-up with reported improvement of symptoms. **(A)** Interval increase in the cavernous sinus mass size with extension into the pituitary fossa. **(B)** Subtle enhancement along V2 on the left, suggesting infiltrative process(inlet). **(C)** Narrowing of the left ICA.

Furthermore, 6 weeks after his initial presentation, follow-up MRI revealed an increase in the size of the cavernous sinus lesion from 1.7 × 1.7 × 1.6 cm to 1.8 × 2.0 × 1.8 cm, demonstrating further extension of the lesion into the Meckel’s cave and along the trigeminal maxillary division, accompanied by further narrowing of the left ICA (Figure 1). He underwent a positron emission tomography (PET) scan to evaluate for other areas of malignancy/granulomatous disease, with the differential diagnoses being unchanged. The patient continued to experience headaches; however, cranial nerve III and VI palsies and diplopia had resolved.

**Figure 2:**
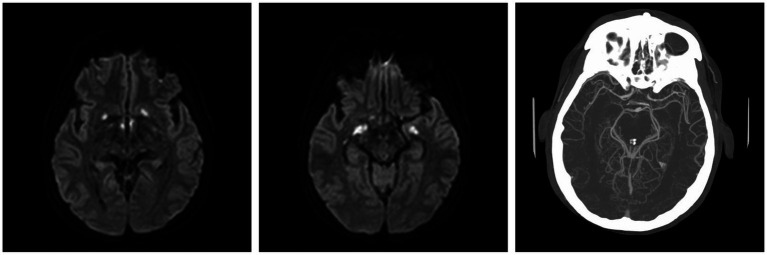
At the ED admission with altered mental status, MRI brain demonstrating basal ganglia and medial temporal infarcts. CTA head demonstrating multifocal stenoses involving bilateral ICA.

**Figure 3:**
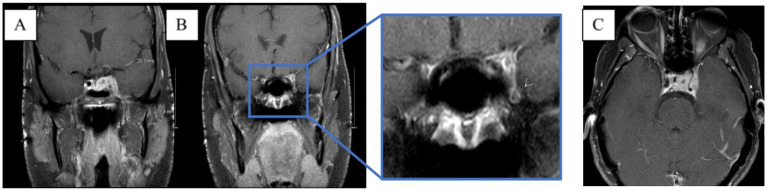
MRI brain T1 + C when the patient lost corneal and pupillary reflexes. Subsequently transferred to the ICU. **(A)** Patchy enhancement within basal ganglia. **(B)** Worsened leptomeningeal enhancement around the suprasellar cistern. **(C)** Non-enhancing cystic focus suggesting thrombus or abscess.

Moreover, 13 weeks after the initial presentation, he returned to the ED with altered mental status and was admitted for evaluation. On examination, he demonstrated word-finding difficulties, impaired attention, and neck stiffness. No other focal signs were noted. MRI re-imaging demonstrated new leptomeningeal enhancement and acute strokes of the bilateral basal ganglia, hypothalamus, and medial temporal lobes, with vascular imaging demonstrating multifocal stenoses with bilateral ICA involvement (Figure 2). The CSF studies revealed significant neutrophilic pleocytosis with an elevated white blood cell count (WBC) of 3,089 uL (72% polymorphonuclear neutrophils (PMNs)), hypoglycorrhachia (35 mg/dL), and elevated protein (124 mg/dL; Table 1). Infectious workup, including bacterial, fungal, and acid-fast cultures; BioFire Meningitis/Encephalitis PCR panel; *Nocardia* cultures; cryptococcal antigen; and Toxoplasma PCR, was negative. Broad-spectrum antimicrobials, including vancomycin, ceftriaxone, ampicillin, acyclovir, and amphotericin, were initiated for empiric meningitis coverage, covering typical and atypical bacterial, viral, and fungal meningitides in the setting of a seemingly indolent course. Ampicillin and acyclovir were quickly discontinued due to negative testing. The patient was tapered off outpatient steroids during hospitalization. Despite serial CSF studies showing an improving cell count (Table 1), symptoms continued to worsen, leading to a comatose state, which resulted in the patient being intubated and transferred to the intensive care unit on hospital day 3. Serial imaging showed new and expanding regions of diffusion restriction involving the basilar artery territory, as well as proximal posterior cerebral artery stenosis presumably secondary to vasospasm (Figure 3). Magnetic resonance angiography of the head showed enhancement around the basilar artery, raising concern for infectious vasculitis.

**Table 1:** CSF values over the disease course, starting from initial presentation to hospitalization 3 months later.

Components of CSF	Initial	Day 75	Day 78	Day 80	Day 82	Day 89
WBC	4 uL	3,089 uL	1,334 uL	312 uL	17 uL	17 uL
PMN	10%	72%	56%	53%	6%	50%
Lymphocytes	86%	24%	40%	46%	91%	45%
Mono/histocytes	4%	4%	4%	1%	3%	5%
RBC	<3,000 uL	<3,000 uL	<3,000 uL	<3,000 uL	<3,000 uL	<3,000 uL
			Karius test			

**Figure 4:**
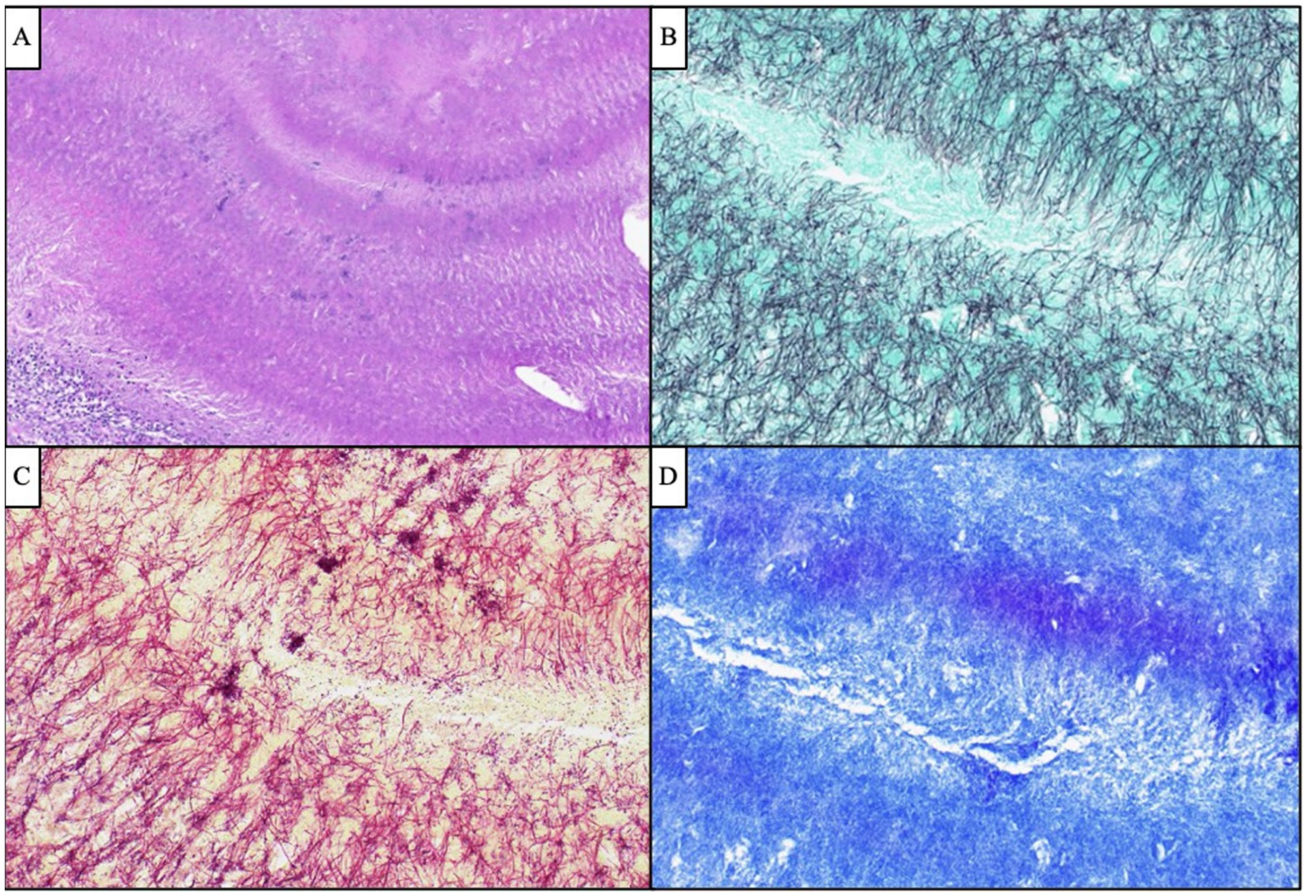
Antemortem lateral skull base endonasal biopsy demonstrated “sulfur granule”-like aggregates of branching filamentous bacteria (**A**, H&E, 100x), which were GMS-positive (**B**, 400x) Gram-indeterminate (**C**, 400x), and FITE-negative (**D**, 400x), compatible with *Actinomyces* species. Scattered and clustered cocci were also visualized by Gram stain (**C**, 400x).

**Figure 5:**
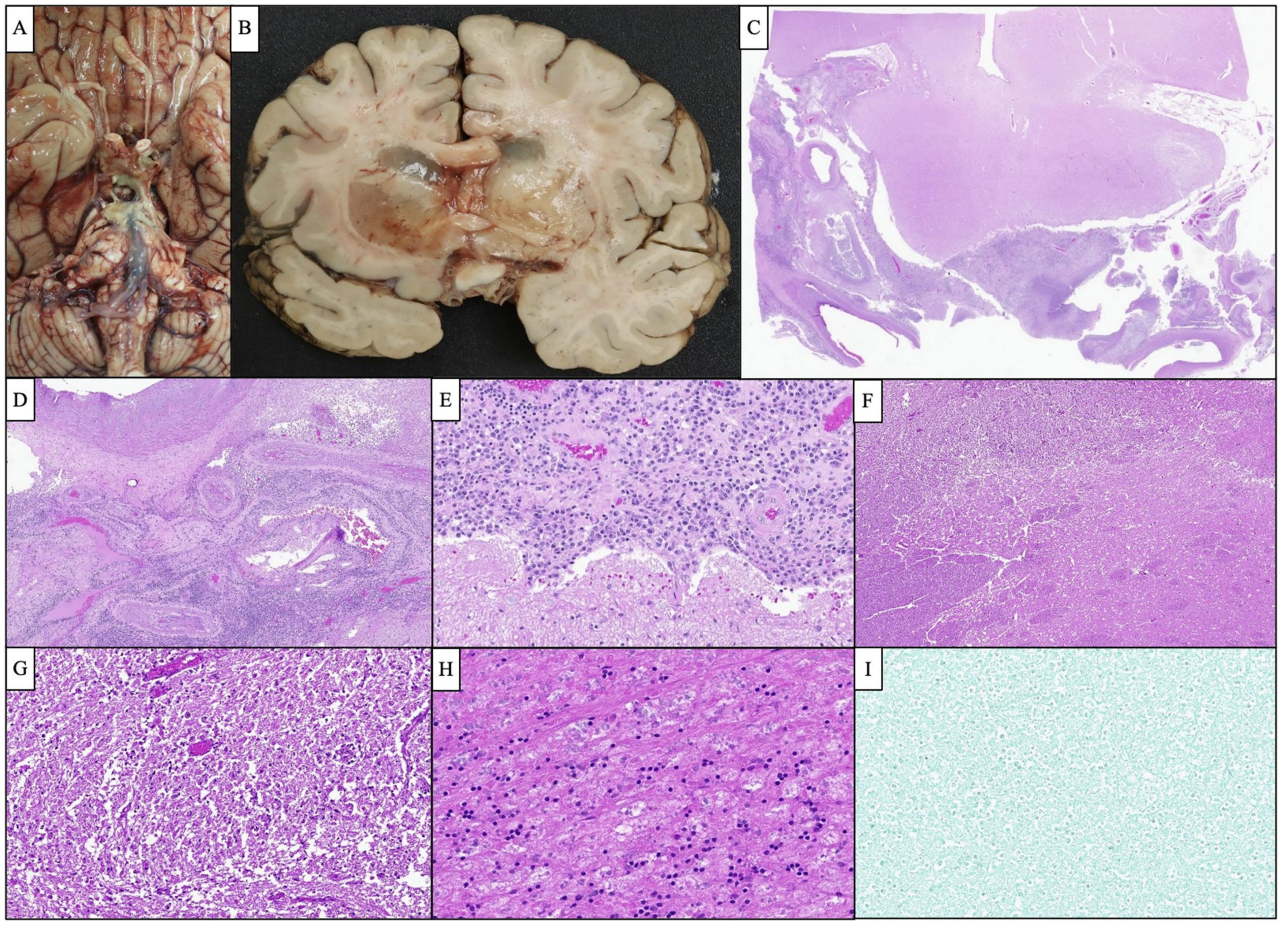
Representative images of the autopsy findings. **(A)** Gross photograph of the brain’s base demonstrated a purulent fluid extending from the optic chiasm inferiorly along the brainstem down to the pons. **(B)** Bilateral basal ganglia showed severe edema and softening and were focally filled with gelatinous material causing marked compression of the adjacent ventricles. **(C)** Microscopic examination of the cavernous sinus revealed dense inflammation and focal abscess formation. **(D)** The inflammatory process extended to the wall of the large caliber vessels. Several smaller vessels appeared occluded with recanalization of the lumen. **(E)** Cavernous sinus inflammation extended to the leptomeninges causing severe meningitis. **(F,G)** Low and high-magnification images of the basal ganglia show severe edema and numerous macrophages consistent with subacute infarction. **(H)** The pituitary gland showed extensive necrosis with focal chronic inflammation. **(I)** GMS silver stain performed on multiple sections was negative for organism growth.

**Figure 6:**
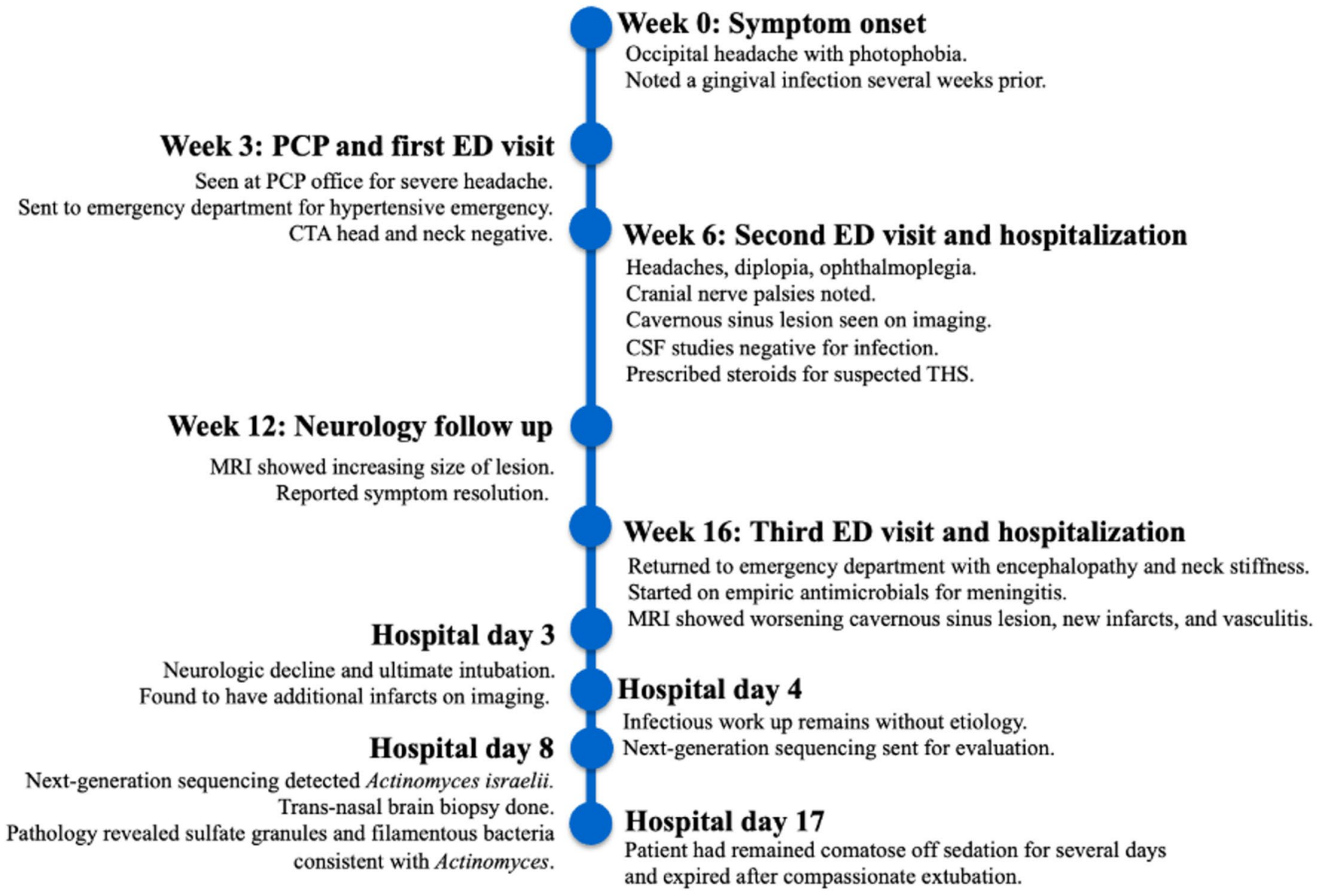
Case report key event timeline.

Given the patient’s worsening clinical symptoms and negative microbiologic workup, a serologic NGS test (Karius Test) was performed, which returned positive for *Actinomyces israelii* at 61 DNA molecules per microliter (MPM) and human herpesvirus 7 (HHV7) at 30 DNA MPM. The pathogenicity of *Actinomyces* was undetermined, while HHV7 was thought to be incidental. To confirm clinical significance, a transsphenoidal skull base incision and drainage were performed, revealing a necrotic-appearing pituitary gland and readily expressed purulent fluid. Pathological examination showed sulfur granule-like aggregates of branching filamentous bacteria consistent with *Actinomyces* (Figure 4). Targeted tissue NGS (Broad-Range PCR) also revealed trace *Actinomyces israelii,* further confirming the diagnosis of CNS actinomycosis. Broad-spectrum antibiotics were narrowed to IV ceftriaxone. Antibiotics were not further narrowed due to the presence of Gram-indeterminate cocci, which were suspected to represent either a secondary bacterial infection or dysmorphic actinomyces. Given the extensive infarcts involving the basal ganglia, thalami, brainstem, and cortices, the family elected to pursue comfort measures. The patient expired shortly after compassionate extubation, approximately 9 days after the suspected diagnosis of CNS actinomycosis. The autopsy revealed diffuse edema and purulence at the skull base, with areas of necrosis and inflammation extending to the walls of the large-caliber vessels. There were regions showing signs of subacute infarcts and meningitis as well. The cause of death was determined to be brain abscess formation with subsequent acute and chronic meningitis (Figure 5). A timeline of the patient’s disease course is provided in Figure 6.

## Methods

Informed consent for publication was obtained from the patient’s mother prior to any literature review or write-up.

MEDLINE (via EBSCOhost), Embase (via embase.com, 1974-present version), the Cochrane Library (via Wiley), and Scopus were searched on 12 November 2023. The searches were composed of keywords and subject headings (when available) for the two search concepts: (1) Tolosa Hunt syndrome and (2) infection, especially infection in the area of the cavernous sinus or involving microorganisms that cause infections in this region. See complete search strategies at https://digitalcommons.unmc.edu/search/22/. Before CS developed the search strategies, CS and NB reviewed search results for articles concerning infectious mimics of Tolosa Hunt or causes of cavernous sinusitis to identify the infectious agents that should be specifically mentioned in the search strategy. There was no patient or public involvement in the design, conduct, reporting, or dissemination of this research.

No language or publication type filters were applied to the searches. Articles indexed as involving animals were removed if they were not also indexed as involving humans. The results of all searches were imported into an EndNote library. After de-duplication in EndNote, the results were imported into Zotero and processed using Zotero’s de-duplication tool. NB performed the title and abstract review.

The inclusion criteria included cases originally diagnosed as THS that were later found to be CNS actinomycosis. THS was diagnosed by the original physician according to the diagnostic criteria and technological capabilities at that time. CNS actinomycosis was defined by biopsy with pathological findings consistent with actinomycosis, such as *Actinomyces* identified on tissue or CSF cultures, or, in cases of disseminated actinomycosis, by the presence of a clinicoradiographically correlated cavernous sinus lesion on imaging with a presumptive diagnosis.

## Results

A total of 593 records were retrieved through the database searches: 218 from Embase, 111 from MEDLINE, one from the Cochrane Library, and 263 from Scopus. A total of 235 duplicates were identified using the EndNote and Zotero duplicate detection tools, and 14 were removed manually during the review. After the removal of the duplicates, 344 records remained for title and abstract screening.

### Patients’ characteristics

This systematic review identified seven additional cases of CNS actinomycosis that mimicked THS (Table 2). The eight identified cases showed a male predominance, with 75% of patients being male. Ages ranged from 6 to 63 years, with a median age of 43. These cases did not seem to be specific to a particular ethnic population and were reported in Italy, Spain, the United Kingdom, Japan, Australia, and the United States.

**Table 2:** Cases of CNS actinomycosis mimicking Tolosa-Hunt syndrome.

Cases	Demographics	Time to initial presentation	Immunocompromised	Initial ESR	Response to steroids	Dental history	Initial CSF	CSF culture	Biopsy culture	Method of ID	Treatment	Outcome
F (19)	F, 27	6 weeks	No	39 (H)	Initial improvement (80 mg prednisone)	No; ear and parotid infections	Acellular	---	-----	Myringotomy culture	Tetracycline (6 wks)	Resolved
G (20)	M, 43	8 weeks	Unknown	----	Initial improvement (30 mg prednisone)	Yes	Pleocytosis (624 cells/mm^3^)	Negative	Negative	Direct microscopic visualization of CNS biopsy	Penicillin G (8 wks) + penicillin V (3 mo)	Resolved
D (14)	M, 43	1 week	No	30 (H)	Initial improvement	No	Acellular	Negative	-----	Direct microscopic visualization of CNS biopsy	Penicillin G (6 wks) + amoxicillin (6 mo)	Resolved
C (21)	F, 53	8 weeks	Yes	(H)	No improvement	Yes	----	----	-----	Direct microscopic visualization of lung biopsy	Penicillin G (42 days every other day) + amoxicillin (4 mo)	Resolved
B (22)	M, 6	4 weeks	No	39 (H)	Not given	Yes	Pleocytosis (74 cell/mm^3^)	Negative	-----	Direct microscopic visualization of CNS biopsy	Penicillin G (6 wks) + pen V (6 wks)	Resolved
A (13)	M, 63	8 weeks	No	81 (H)	Initial improvement	Yes	1 mononuclear cell/mm^3^	Negative	Negative	Direct microscopic visualization of CNS biopsy	Penicillin (6 wks) + amoxicillin (1 year)	CNV1 hypoaesthesia and mild right ptosis
E (23)	M, 57	3 weeks	No	48 (H)	No improvement	No; extractions after symptoms	Acellular	Negative	-----	Direct microscopic visualization of autopsy	None	Stroke, death
H (case report above)	M, 43	3 weeks	No	44 (H)	Initial improvement	Yes	4 WBC/mm^3^	Negative	Negative	Direct microscopic visualization of CNS biopsy and Karius testing	Broad-spectrum (3 wks)	Stroke, death

### Clinical characteristics

Each patient presented with eye pain and diplopia, with or without headaches, aligning with the THS diagnostic triad. The average time to presentation was 5.1 weeks, ranging from 1 to 8 weeks. A total of seven of the eight cases were localized to the cervicofacial region. Furthermore, one patient had disseminated actinomycosis and was on immunosuppressive medications, while five patients had dental infections or extractions prior to symptom onset, and one had extractions after symptom onset. Moreover, one case was associated with otitis media, while five cases had a fever on presentation or during hospitalization. Pleocytosis was noted in three of the cases, each occurring weeks to months after initial presentation. CSF cultures were obtained in 75% of the cases, all of which were negative. In addition, three cases reported culturing of biopsied tissue, each returning negative. In seven of the eight cases, the diagnosis was made by histopathologic examination, consistent with *Actinomyces,* through direct visualization of branching filaments, with or without the presence of sulfur granules. The biopsy sources were the cavernous sinus (*n* = 6) and the lung (*n* = 1). The only case to successfully culture *Actinomyces* obtained the cultured specimen via myringotomy. No cases reported isolation of *Actinomyces* from blood cultures.

### Treatment/outcome

In total, five cases were treated with IV penicillin for a duration of 6–8 weeks, followed by oral penicillin/amoxicillin for 6–52 weeks. In addition, one case of disseminated actinomycosis received IV penicillin every other day for 42 days, followed by 16 weeks of amoxicillin, while one case was treated with 6 weeks of tetracycline. Another case received broad-spectrum antibiotics prior to expiration. Furthermore, five cases made a full recovery, while one reported CNV1 hypoesthesia and mild right ptosis. The patients in the remaining two cases passed away. Each of these cases was complicated by vasculitis, multiple acute infarcts, and coma. The autopsies showed similar abscesses in the cavernous sinus with purulent material. Of additional importance, 62.5% of the cases saw initial symptom improvement upon starting steroids. This is important because it can create a false sense of security regarding the accuracy of the diagnosis.

## Discussion

The above case and subsequent review offer several lessons to practitioners who may encounter infections of the head and neck as part of their daily practice.

Firstly, actinomycosis, while easy to treat in its initial stages, remains a diagnostic challenge due to its rarity, indolent growth, and poor sensitivity of standard culture-based testing. Isolation of *Actinomyces* via culture is often unsuccessful (11). Previous literature has reported CNS biopsy culture positivity rates ranging from 0 to 53% (2, 12), and a CSF culture positivity rate of 17% (2). For these reasons, practitioners may prematurely exclude infectious etiologies such as actinomycosis from the differential diagnosis and attribute symptoms to another etiology or a diagnosis of exclusion such as THS. We argue that unremarkable CSF studies are not enough to rule out *Actinomyces* in cases of cavernous sinus disease. We recommend incorporating early biopsy into the THS diagnostic criteria suggested by previous case reports and systematic reviews (10, 13, 14). Early biopsy at symptom onset or during the first hospitalization would have had the potential to save that patient’s life. Even empiric antibiotic therapy with coverage for A*ctinomyces* proved ineffective. In addition, we advocate for biopsied tissue to be also submitted for histologic examination by pathology, rather than culture alone.

Secondly, the current diagnostic criteria for THS should be used with extreme caution. THS is defined by the ICHD-3 as unilateral orbital/periorbital pain with associated paresis of cranial nerves III, IV, and/or VI and granulomatous inflammation of the cavernous sinus, superior orbital fissure, or orbit. These diagnostic criteria have previously been criticized for leading to premature diagnosis and inappropriate empiric corticosteroid treatment (15, 16). This is exacerbated by the comments provided with the ICHD-3 criteria, in which monitoring for alternative diagnoses is encouraged merely on close follow-up rather than preemptively. In addition to delaying care, steroid treatment may aggravate infectious etiologies in the long term, leading to worse outcomes. CNS actinomycosis has an overall fatality rate of 11%, with an additional 22% of patients having residual deficits (2). In comparison, our case review revealed a fatality rate of 28.5%, supporting the hypothesis that the current ICHD-3 THS diagnostic criteria may hinder the accurate diagnosis of CNS actinomycosis, thereby increasing the risk of significantly higher mortality. We strongly recommend that future editions of the ICHD revise the THS diagnostic criteria to explicitly require an extensive diagnostic work-up, especially in cases of symptom recurrence, and to adopt a more restrictive approach to diagnosing THS.

Finally, organism identification by NGS is a relatively new and rapidly growing area of clinical microbiology. NGS has several methods, including metagenomic NGS (mNGS), targeted NGS (tNGS), and whole genomic sequencing (WGS). The Karius Test® is an mNGS test that detects microbial cell-free DNA in blood. It can detect an organism’s nucleic acids without target-specific primers, which allows for the identification of more than a thousand bacteria, fungi, parasites, and DNA viruses without prior input from medical staff (17). Karius testing is believed to identify organisms in CSF due to the leakage of infectious material across the blood–brain barrier during inflammation. It has been found to have a sensitivity of 92.9% for blood-borne infections (17); however, its sensitivity in CNS infections is variable. In a systematic review and meta-analysis of 12 studies assessing the diagnostic accuracy of mNGS of CSF, sensitivity was found to be approximately 77%, with a specificity of approximately 96% (18). One challenge with NGS testing is that it only identifies the presence of an organism’s DNA in a sample and not whether it is the pathogen of interest. Since many organisms are part of the normal flora in various areas of the body, it should be followed up with confirmatory testing. Expert opinion encourages NGS to be conducted as an adjunct rather than a replacement for traditional testing methods. It may have utility in specific scenarios, such as cases where infection is suspected but culture-based testing remains negative (17). In our case, because *Actinomyces* colonizes the oropharynx, it can be difficult to determine if it is the pathogen of interest, thereby requiring a biopsy for confirmation. However, we propose that NGS may be beneficial as an alternative to biopsy in cases with anatomic difficulties and/or patient preference. In addition, NGS can complement traditional infectious work-ups in complicated cases, allowing for judicious broadening of antibiotic coverage.

## Conclusion

Given the low reported prevalence of CNS actinomycosis, the inadequacy of current testing for *Actinomyces*, and the significant overlap of symptoms with THS, it is essential that clinicians are aware of CNS actinomycosis as a potential infectious etiology to provide early and potentially curative treatment. The current definition of THS, as published in the ICHD-3, may encourage harmful practices and should be revised. Further research is needed to improve cultural sensitivities and next-generation sequencing techniques for *Actinomyces* detection.

## Data Availability

The original contributions presented in the study are included in the article/supplementary material, further inquiries can be directed to the corresponding authors.
